# Do E_2_ and P_4_ contribute to the explained variance in core temperature response for trained women during exertional heat stress when metabolic rates are very high?

**DOI:** 10.1007/s00421-022-04996-2

**Published:** 2022-07-07

**Authors:** Huixin Zheng, Claire E. Badenhorst, Tze-Huan Lei, Ahmad Munir Che Muhamed, Yi-Hung Liao, Naoto Fujii, Narihiko Kondo, Toby Mündel

**Affiliations:** 1 School of Sport Exercise and Nutrition, Massey University, Palmerston North, New Zealand; 2 School of Sport Exercise, Nutrition, Massey University, Auckland, New Zealand; 3 College of Physical Education, Hubei Normal University, Huangshi, China; 4 Advanced Medical and Dental Institute, Universiti Sains Malaysia, Kepala Batas, Malaysia; 5 Department of Exercise and Health Science, National Taipei University of Nursing and Health Sciences, Taipei, Taiwan; 6 Faculty of Health and Sport Sciences, University of Tsukuba, Tsukuba, Japan; 7 Laboratory for Applied Human Physiology, Graduate School of Human Development and Environment, Kobe University, Kobe, Japan

**Keywords:** Body temperature, Regression, Performance, Exercise, Estrogen, Females

## Abstract

**Purpose:**

Women remain underrepresented in the exercise thermoregulation literature despite their participation in leisure-time and occupational physical activity in heat-stressful environments continuing to increase. Here, we determined the relative contribution of the primary ovarian hormones (estrogen [E_2_] and progesterone [P_4_]) alongside other morphological (e.g., body mass), physiological (e.g., sweat rates), functional (e.g., aerobic fitness) and environmental (e.g., vapor pressure) factors in explaining the individual variation in core temperature responses for trained women working at very high metabolic rates, specifically peak core temperature (*T*_peak_) and work output (mean power output).

**Methods:**

Thirty-six trained women (32 ± 9 year, 53 ± 9 ml·kg^−1^·min^−1^), distinguished by intra-participant (early follicular and mid-luteal phases) or inter-participant (ovulatory vs. anovulatory vs. oral contraceptive pill user) differences in their endogenous E_2_ and P_4_ concentrations, completed a self-paced 30-min cycling work trial in warm–dry (2.2 ± 0.2 kPa, 34.1 ± 0.2 °C, 41.4 ± 3.4% RH) and/or warm–humid (3.4 ± 0.1 kPa, 30.2 ± 1.2 °C, 79.8 ± 3.7% RH) conditions that yielded 115 separate trials. Stepwise linear regression was used to explain the variance of the dependent variables.

**Results:**

Models were able to account for 60% of the variance in *T*_peak_ ($\overline{R }$^2^: 41% core temperature at the start of work trial, $\overline{R }$^2^: 15% power output, $\overline{R }$^2^: 4% [E_2_]) and 44% of the variance in mean power output ($\overline{R }$^2^: 35% peak aerobic power, $\overline{R }$^2^: 9% perceived exertion).

**Conclusion:**

E_2_ contributes a small amount toward the core temperature response in trained women, whereby starting core temperature and peak aerobic power explain the greatest variance in *T*_peak_ and work output, respectively.

## Introduction

Determining the factors that influence the female response to exertional heat stress is not new (Nunneley 1978; Stephenson and Kolka 1993), although different research approaches have been employed. One approach compares *differences in the group mean* with that of an intervention or other matched group when all characteristics apart from the one under investigation are standardized (Gagnon and Kenny 2012; Charkoudian and Stachenfeld 2014). Another approach considers the *relative contribution of independent variables* in explaining a dependent variable from individual responses of a (usually larger) heterogenous sample, seen as a better representation of the population distribution (Foster et al. 2020). Concerning the latter, previous studies (Havenith et al. 1998; Notley et al. 2019) with the largest number of recreationally active women (*n* = 36 and 43, respectively) have sought to determine thermoregulatory responses to low–moderate fixed-intensity cycle ergometry for 30- to 60-min bouts measured in a range of ambient conditions (from temperate to warm–humid and hot–dry). Both studies used regression analysis to determine which morphological (body mass, surface area and % fat, etc.), physiological (metabolic rate or heat production, whole-body or local sweat rates, etc.), functional (aerobic fitness and power) and environmental (ambient temperature and absolute humidity) factors explained the variance in the women’s’ core temperature (*T*_core_) response. Results indicated that the strength of the relationships and variance explained (10–59%) was dependent on the heat load, i.e., combined exercise intensity and ambient thermal profile of the trials (Havenith et al. 1998; Notley et al. 2019). While these important results are valid for occupational and leisure-time physical activity completed at a low–moderate intensity (or metabolic rates), they are unlikely to be representative of or applicable to aerobically trained women undertaking such activities at higher intensities for a number of reasons.

Firstly, metabolic heat production in trained women at these higher intensities is likely double the values previously examined in the literature, i.e., metabolic rates of 148–389 vs. 464–716 W·m^−2^ (Lei et al. 2019; Notley et al. 2019), while trained women have a greater capacity to deal with a heat load on account of their enhanced heat loss effectors (Kuwahara et al. 2005). Next, these previous studies have not reported or accounted for differences in thermoregulation secondary to fluctuations in the primary ovarian steroids (E_2_ and P_4_), whereby generally speaking E_2_ promotes heat dissipation and lowers *T*_core_, while P_4_ has the opposite effect (Charkoudian and Stachenfeld 2014). This is important to consider as this may differ from less trained counterparts (Kuwahara et al. 2005) and has been shown to contribute to the variance in *T*_core_ at rest (Lei et al. 2017). Finally, the nature of a fixed-intensity protocol denies the user of behavioral thermoregulation (Schlader et al. 2011a), thereby ignoring the fundamental premise that heat loss needs only to equal heat production (Nielsen 1938) and is considered to be less ecologically valid (than self-pacing) for most leisure-time and occupational physical activity apart from few, i.e., forced marching.

The purpose of the current paper was to determine the relative contribution of the E_2_ and P_4_ alongside other morphological, physiological, functional and environmental factors in explaining the individual variation in trained women when considering the core temperature response (peak *T*_core_, [*T*_peak_]) and work output (mean power output) with very high metabolic rates. To achieve this, we retrospectively analyzed results from 36 trained women completing a self-paced 30-min work trial that has been shown to be unaffected by ovulatory status, ambient environment and pre-load/warm-up duration (Zheng et al. 2021b). Participants were distinguished by intra-participant (i.e., early follicular and mid-luteal phases) or inter-participant (i.e., ovulatory vs. anovulatory vs. oral contraceptive pill [OCP] user) differences in their endogenous E_2_ and P_4_ concentrations. We hypothesized that in addition to previously identified factors such as body mass, aerobic fitness and metabolic heat production (Havenith et al. 1998; Notley et al. 2019), the ovarian hormones would contribute significantly toward the variance explained in *T*_core_ during exercise.

## Methods

This paper combines data from three separate experiments (Lei et al. 2017, 2019; Zheng et al. 2021a), which included *n* = 28 ovulatory and OCP-user female cyclists/triathletes and adds to this new data of the *n* = 8 participants that did not complete all trials or were excluded from the final analyses on account of being deemed anovulatory (Lei et al. 2017; Zheng et al. 2021a). Interested readers are directed to these studies for further methodological details and results.

## Ethical approval

All original studies (Lei et al. 2017, 2019; Zheng et al. 2021a) had received approval by the Massey University Human Ethics Committee (Southern A) and were performed in accordance with the latest revision of the *Declaration of Helsinki*, except for registration in a database. Informed, written consent was obtained from all participants prior to their participation.

## Participants

**Fig. 1:**
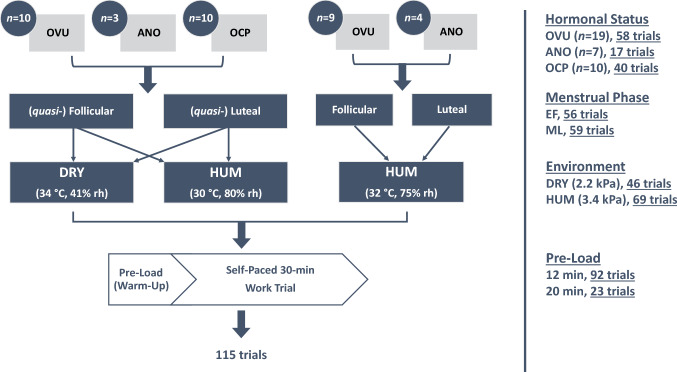
Diagram of experimental overview. Ovulatory (OVU), anovulatory (ANO) and oral contraceptive pill (OCP) users performed trials in their (*quasi*-) early follicular (EF) and/or mid-luteal (ML) phases in warm–dry (DRY) and/or warm–humid (HUM) environmental heat. *n* = 23 completed four trials and *n* = 10 completed two trials, whereas *n* = 3 completed only one trial due to scheduling difficulties and dropout

Thirty-six aerobically trained women participated, yielding 115 separate trials (*n* = 23 completed 4 trials, *n* = 10 completed 2 trials, *n* = 3 completed 1 trial, see Fig. 1). Their physical characteristics are displayed in Table 1. Inclusion criteria were that participants were healthy non-smokers not taking any regular medication (apart from those using the OCP), cycling regularly (≥ 3 days per week) with a maximal aerobic capacity (VO_2_max) ≥ 40 ml·kg^−1^·min^−1^. Exclusion criteria included any cardiovascular, metabolic, neurological and respiratory diseases. All eumenorrheic women self-reported a regular menstrual cycle 21–35 days in length (≥ 3 month) with no use of hormonal contraception (≥ 6 mo). All OCP women were taking a monophasic combination OCP (≥ 1 year) with experimental visits completed during the 3 weeks of active pill use (see Lei et al. 2019 for further details).

**Table 1:** Participant characteristics for ovulatory (OVU), anovulatory (ANO) and oral contraceptive pill (OCP) groups

Characteristic	OVU (*n* = 19)	ANO (*n* = 7)	OCP (*n* = 10)	Mean (*n* = 36)	*p* value
Age (y)	34 ± 9 (19–46)	37 ± 10 (22–51)	25 ± 5 (20–36)*	32 ± 9 (19–51)	0.01
Mass (kg)	63 ± 6 (46–69)	60 ± 7 (46–69)	68 ± 10 (58–82)	64 ± 8 (46–82)	0.13
A_D_ (m^2^)	1.70 ± 0.11 (1.49–1.94)	1.63 ± 0.12 (1.37–1.72)	1.76 ± 0.13 (1.60–1.98)	1.70 ± 0.12 (1.37–1.98)	0.12
A_D_: mass	0.027 ± 0.001 (0.024–0.029)	0.027 ± 0.002 (0.025–0.030)	0.026 ± 0.002 (0.023–0.028)	0.027 ± 0.002 (0.023–0.030)	0.37
% fat	23 ± 5 (15–37)	20 ± 5 (13–29)	24 ± 5 (16–32)	23 ± 6 (13–37)	0.19
VO_2_max (L·min^−1^)	3.3 ± 0.6 (2.3–4.6)	3.4 ± 0.9 (2.7–5.0)	3.7 ± 0.5 (3.0–4.8)	3.4 ± 0.6 (2.3–5.0)	0.22
PPO (W)	270 ± 40 (225–392)	283 ± 31 (250–325)	283 ± 29 (248–325)	276 ± 35 (225–392)	0.56
Training history (y)	7.1 ± 3.5 (4–16)	8.1 ± 5.1 (1–15)	3.7 ± 2.5 (2–10)*	6.3 ± 3.9 (1–16)	0.03

Values are mean ± SD (range)

*A*_*D*_ Du Bois body surface area, *PPO* peak aerobic power, *VO*_*2*_*max* maximal rate of O_2_ consumption

^*^Significantly different from both other groups

## Ovulatory status and ambient conditions

Eumenorrheic women were tested on days 3–6 (EF) and 18–21 (ML) following the *start of menses*, while OCP women were tested on days 3–6 and 18–21 following the *start of active OCP use*. Our rationale for comparing EF and ML was based on maximizing the differences in E_2_ and P_4_ occurring naturally, permitting comparison with/expansion beyond previous results, and that ovulatory women are in EF and ML for ~ 50% of their reproductive lives. Although this approach represents the phases of lowest hormone exposure and peak P_4_, it does not include for comparison the late-follicular/pre-ovulatory phase. Although the late-follicular/pre-ovulatory phase captures when E_2_ peaks, the duration of < 72 h makes it difficult to perform repeated tests (such as this study) and comprises a much smaller proportion of the reproductive life for these women. Testing for eumenorrheic women was scheduled using the three-step method (Allen et al. 2016) whereby self-reported menses onset and urinary luteinizing hormone testing (EasyCheck® Ovulation Test, Phoenix Medcare Ltd, Auckland, New Zealand) prospectively identified EF and ML, while measurement of serum 17β-estradiol (E_2_) and P_4_ retrospectively confirmed ML. A P_4_ level of > 5 ng·ml^−1^ is good evidence that ovulation has occurred (Leiva et al. 2015; Schaumberg et al. 2017; Scheid and De Souza 2010). Therefore, participants were deemed as ovulatory (OVU, > 5 ng·ml^−1^) or anovulatory (ANO, < 5 ng·ml^−1^) as detection of a urinary luteinizing hormone surge (alone) cannot confirm luteal phase sufficiency (Scheid and De Souza 2010). Ambient conditions were distinguished by vapor pressure, such that the following characterized each environment: DRY (2.2 ± 0.2 kPa, 34.1 ± 0.2 °C, 41.4 ± 3.4% RH, wet-bulb globe temperature: 27.0 ± 0.5 °C) and HUM (3.4 ± 0.1 kPa, 30.2 ± 1.2 °C, 79.8 ± 3.7% RH, wet-bulb globe temperature: 28.2 ± 0.8 °C).

## Experimental overview

All data were collected outside of the Southern Hemisphere summer (March–November) where the average daily temperature did not exceed 22 °C, nor had participants spent any time in a warmer climate for at least 1 month prior to the study. All participants attended the laboratory on the following occasions: (1) preliminary submaximal and maximal aerobic capacity test, (2) experimental familiarization and (3) experimental trials. For an overview of the experimental design see Fig. 1. The experimental trials consisted of the following factors: (*quasi*-) menstrual phase (early follicular [EF, *56 trials*] and mid-luteal [ML, *59 trials*]) and ambient profile (warm–-humid [HUM, *69 trials*] and warm–dry [DRY, *46 trials*]). The order of the trials was randomized and counterbalanced except the order of the ambient profile was consistent in different (*quasi*-) phases within participants. Experimental trials were conducted at the same time of the morning (± 1 h) and following > 24 h of dietary and exercise control. Each trial consisted of either 12 or 20 min of fixed-intensity pre-load that was kept consistent within participants, immediately followed by a 30 min of self-paced work trial where only percentage of time elapsed (every 20% or 6 min) was provided to the participant. All exercise was performed on an electronically braked cycle ergometer (Lode Excalibur, Groningen, The Netherlands), with handlebars, seat height and pedal preference standardized according to individual preference. The typical timeline for a participant to complete this study resulted in preliminary testing and familiarization separated by 3–7 days during the (*quasi*-) follicular phase, with half of the participants starting their experimental trials the following (*quasi*-) luteal phase (i.e., 14 days later) and the other half the following (*quasi*-) follicular phase (i.e., 28 days later), with within-phase experimental trials differing by ambient profile separated by 3 days.

## Preliminary testing and familiarization

All preliminary testing was conducted in the (*quasi*-) EF phase of each participant’s menstrual cycle to minimize the potential effects of menstrual/OCP cycle on their physiological and performance responses during the tests (Sims and Heather 2018). Following anthropometric measurements (height, weight, body composition), a 24-min steady-state submaximal cycle ergometer test was conducted in a temperate laboratory environment (18–22 °C) with a fan-generated airflow of 19 km·h^−1^ facing participants. The submaximal cycle test consisted of four consecutive 6-min stages with power outputs of 100 W, 125 W, 150 W and 175 W at comfortable, but constant cadence. O_2_ consumption was measured during the last 2 min of each stage. Following 10-min rest from the submaximal test, a VO_2_max cycle ergometry test was performed. The initial workload began at 100 W and increased by 25 W every minute, until volitional exhaustion. The exercise intensity during the self-paced exercise was based on 75% of an individual’s VO_2_max, which was derived from the linear relationship between the power output and the O_2_ consumption during both the steady-state submaximal exercise test and maximal aerobic capacity test. Following at least 24 h rest from the preliminary session, a familiarization trial was conducted to ensure all participants were familiar with the testing procedures and to minimize the learning effect during trials. This trial was replicated entirely during the experimental trials outlined below.

## Dietary and exercise control

Diet and physical activity during the 48 h prior to the first experimental trial were recorded and participants were instructed to repeat these for the following experimental trials. The day of and prior to any experimental trial was marked by abstinence from alcohol, exercise and only habitual caffeine use (as abstinence would confound results from withdrawal effects). This dietary and exercise control minimized variation in pre-trial metabolic state. Fluid intake was encouraged to ensure a euhydrated state.

## Experimental procedure

These trials were conducted in the same environmental chamber with a fan-generated airflow of 19 km·h^−1^. Upon their arrival at the laboratory, participants voided, producing a urine sample to confirm a urine specific gravity < 1.020 to ensure adequate hydration (Sawka et al. 2007). Following this, nude body weight was recorded and participants self-inserted a rectal thermistor 12 cm beyond their anal sphincter. A blood sample was obtained from an antecubital vein after participants had rested seated for 15 min. Participants entered the environmental chamber wearing only cycling shorts and top, shoes and socks. Participants rested seated on the ergometer for 20 min during which they were instrumented, and baseline measurements were recorded. They then completed either i) 6 min of cycling at each of 125 and 150 W (62 ± 9 and 73 ± 10% VO_2_max, respectively, *92 trials*) or ii) 10 min of cycling at each of 100 and 125 W (56 ± 8 and 68 ± 10% VO_2_max, respectively, *23 trials*); notably, where participants completed multiple trials, the warm-up duration was kept constant. Physiological measurements taken during the final 2 min of each intensity included expired gas and rating of perceived exertion RPE, while rectal temperature (*T*_rec_) was measured continuously. Immediately on completion of the second fixed-intensity bout, the ergometer was set to linear mode based on the formula of Jeukendrup et al. (1996), where participants were instructed to perform as much work as possible over 30 min. During this 30-min self-paced period, work completed (kJ) and RPE were recorded every 6 min, while *T*_rec_ was measured continuously and tap water at 20 °C was provided to drink ad libitum throughout to minimize dehydration. Total work completed (kJ) was used as criterion measure for performance, although this was expressed as mean power output for the trial to allow wider application. After the completion of the 30-min self-paced exercise, the participant towel dried and recorded nude body weight.

## Measurements

Results reported in the current study were those for which a maximal number of measures were recorded for the *n* = 36. For interested readers, other physiological (i.e., thermoregulatory, cardiovascular, inflammatory) and reliability measurements were performed during these trials and can be found in our separate studies (Lei et al. 2017, 2019; Zheng et al. 2021a, 2021b).

### Anthropometric

Participant height and weight were measured using a stadiometer (Seca, Germany; accurate to 0.1 cm) and scale (Jadever, Taiwan; accurate to 0.01 kg), from which surface area (A_D_) was estimated (Du Bois and Du Bois 1916). Body composition was measured using multi-frequency bioelectrical impedance analysis (InBody 230, Korea) using a standard procedure (Kyle et al. 2004).

### Respiratory

Expired respiratory gases were collected from a mixing chamber and analyzed for O_2_ consumption using an online, breath-by-breath system (VacuMed Vista,Turbofit, Ventura, CA, USA) using a 30-s average. This system was calibrated before each trial using a zero and *β*-standard gas concentrations, and volume (VacuMed 3L Calibration Syringe).

### Body temperature and sweat loss

*T*_core_ was indexed from *T*_rec_ measured with a rectal thermistor (Covidien Mon-a-Therm, USA; accurate to 0.1 °C) and recorded continuously using TracerDAQ software (Measurement Computing Corporation, Norton, MA, USA). Whole-body sweat rate (WBSR) was estimated from nude body mass loss, corrected for fluid consumed and time.

### Hormones

Venous blood was collected by venipuncture into a vacutainer (Becton–Dickinson, Oxford, UK) containing clot activator and once clotted (> 30 min) the whole blood was centrifuged at 4 °C and 805*g* for 15 min and aliquots of serum were transferred into Eppendorf tubes (Genuine Axygen Quality, USA) and stored at − 80 °C until further analysis. Serum samples were analyzed using enzyme-linked immune assays for E_2_ (Demeditec Diagnostics, Kiel, Germany) and P_4_ (IBL International, Hamburg, Germany) with a sensitivity of 6.2 pg·ml^−1^ and 0.045 ng·ml^−1^, respectively, and an intra-assay variation of < 6 and < 7%, respectively.

### Perceived exertion

RPE was measured using the 15-grade scale, from 6 to 20 (Borg 1970).

### Data and statistical analyses

The dependent variables were mean power output and *T*_peak_. The independent variables included: age, mass, A_D_, mass:A_D_, % body fat, aerobic fitness, peak aerobic power, training history, E_2,_ and P_4_, P_4_:E_2,_ *T*_core_ at baseline (*T*_base_), *T*_core_ at start of work trial (*T*_0_), WBSR, vapor pressure and power output.

All statistical analyses were performed with SPSS software for Windows (IBM SPSS Statistics 25, NY, USA). Descriptive values were obtained and reported as means and standard deviation (± SD). Data were checked for normality by calculating skewness and kurtosis, whereby values within ± 2 were deemed to be acceptable (Weir and Vincent 2021). Participant characteristics were analyzed using one-way ANOVA and Student’s *t* test. Correlation coefficients were calculated to reveal the direction and strength of any potential relationships between variables; Pearson’s correlation coefficient and Spearman's rho were determined for data that did or did not (E_2_, P_4_, P_4_:E_2_) follow a normal distribution, respectively. Finally, in line with and to allow comparison to previous research (Havenith et al. 1998; Notley et al. 2019), stepwise linear regression was used to explain the variance of the dependent variables. A total of 104 (*T*_peak_) and 103 (power output) cases were included for the regression (not 115, due to missing E_2_, P_4_ and sweat rate data), where data that did not follow a normal distribution (E_2_, P_4_, P_4_:E_2_) were log-transformed before entering. Independent variables were only included in the final models if their tolerance value was > 0.5 to avoid unacceptable collinearity between predictors. Data were screened for influential cases using Cook’s distances, leverage values and standardized residuals. Test assumptions for normality, linearity and homoscedasticity were determined by scatter and residual plots. Since some participants completed repeated trials, residuals from each final regression model were tested for serial correlation using the Durbin–Watson test, whereby a value between 1.5 and 2.5 was deemed acceptable (Durbin and Watson 1950). Statistical significance was set at *p* ≤ 0.05.

## Results

As can be seen from Table 2, a wide range of intra- and inter-participant endogenous concentrations in E_2_ and P_4_ was evident. By contrast, other dependent and independent variables displayed far less variability between participants, (*quasi*-) menstrual phases and ambient environments (Table 3).

**Table 2:** Participant hormone concentrations

	(*quasi*-) Follicular						(*quasi*-) Luteal					
	Warm–humid			Warm–dry			Warm–humid			Warm–dry		
	OVU	ANO	OCP	OVU	ANO	OCP	OVU	ANO	OCP	OVU	ANO	OCP
E_2_ (pg·ml^−1^)	63 ± 60 (2–255)	46 ± 14 (36–56)	19 ± 23 (1–75)	55 ± 43 (9–137)	24 ± n.d (−)	17 ± 25 (0.2–79)	108 ± 70 (45–297)	156 ± 128 (42–386)	21 ± 31 (0–102)	86 ± 76 (15–288)	44 ± 7 (39–48)	20 ± 28 (0–89)
P_4_ (ng·ml^−1^)	0.6 ± 0.4 (0.1–1.2)	0.2 ± 0.1 (0.1–0.3)	0.2 ± 0.2 (0.0–0.4)	0.5 ± 0.4 (0.1–1.1)	0.2 ± 0.1 (0.1–0.2)	0.1 ± 0.1 (0.01–0.4)	16.4 ± 10.0 (6.5–52.7)	1.8 ± 2.9 (0.1–7.9)	0.2 ± 0.2 (0.02–0.5)	17.1 ± 18.8 (5.5–69)	0.6 ± 0.6 (0.2–1.3)	0.1 ± 0.1 (0.01–0.5)
P_4_:E_2_	47 ± 150 (1–667)	5 ± 2 (4–6)	28 ± 42 (2–130)	15 ± 14 (2–36)	5.5 ± n.d. (−)	31 ± 59 (3–185)	184 ± 98 (44–415)	23 ± 45 (1–114)	16 ± 11 (4–34)	276 ± 300 (88–1048)	16 ± 15 (5–26)	12 ± 13 (5–44)

*n.d.* SD was not determined due to missing data, *E*_*2*_ 17-β estradiol, *P*_*4*_ progesterone, *P*_*4*_*:E*_*2*_ ratio of progesterone to 17-β estradiol, *OVU* ovulatory group, *ANO* anovulatory group, *OCP* oral contraceptive user group, values are mean ± SD (range)

**Table 3:** Descriptive statistics for dependent and independent variables

	(*quasi*-) Follicular		(*quasi*-) Luteal	
	Warm–humid	Warm–dry	Warm–humid	Warm–dry
Independent variables				
* T*_base_ (°C)	37.2 ± 0.3 (36.6–37.8)	37.3 ± 0.3 (36.8–37.8)	37.4 ± 0.3 (36.6–37.8)	37.4 ± 0.2 (36.9–37.7)
* T*_0_ (°C)	37.7 ± 0.3 (37.1–38.2)	37.8 ± 0.3 (37.4–38.4)	37.9 ± 0.3 (37.1–38.3)	37.8 ± 0.2 (37.6–38.3)
Sweat rate (kg·h^−1^)	0.8 ± 0.3 (0.4–1.6)	0.9 ± 0.2 (0.4–1.3)	0.8 ± 0.3 (0.4–1.9)	0.9 ± 0.4 (0.2–1.8)
RPE	15.1 ± 1.5 (12.6–17.6)	15.5 ± 1.7 (12.3–18.2)	15.2 ± 1.7 (11.8–19.8)	15.5 ± 1.7 (12.4–18.2)
Absolute humidity (kPa)	3.4 ± 0.1 (3.2–3.6)	2.2 ± 0.2 (1.8–2.6)	3.4 ± 0.1 (3.2–3.6)	2.2 ± 0.2 (1.9–2.6)
Dependent variables				
Power output (Watt)	147 ± 29 (90–240)	149 ± 19 (116–192)	144 ± 24 (90–208)	150 ± 20 (98–191)
* T*_peak_ (°C)	38.6 ± 0.3 (37.6–39.2)	38.7 ± 0.3 (38.0–39.4)	38.7 ± 0.4 (37.9–39.6)	38.7 ± 0.3 (38.1–39.3)

Values are mean ± SD (range)

*RPE* rating of perceived exertion, *T*_0_ *T*_core_ at start of work trial, *T*_base_ *T*_core_ at baseline, *T*_peak_ peak *T*_core_

### *T*_peak_

**Fig. 2:**
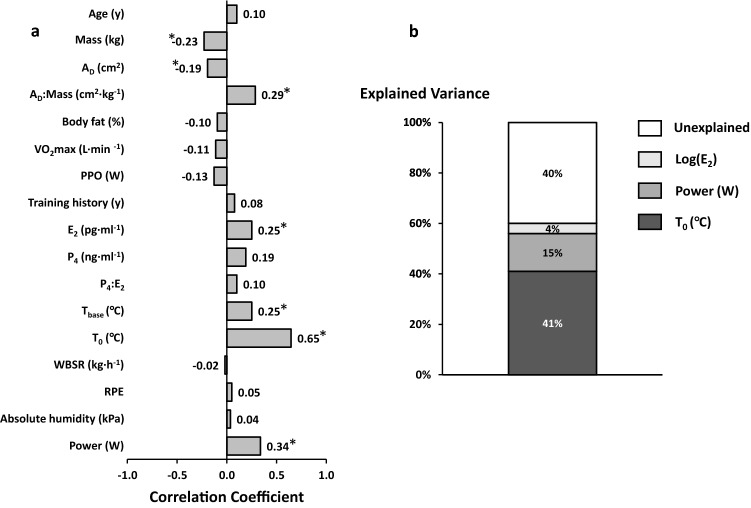
**a** Bivariate associations between independent variables and peak *T*_core_ (*T*_peak_) on all common data points. *p < 0.05. **b** The percentage of explained and unexplained (residual) variance ($\overline{R }$^2^) for explaining *T*_peak_

Correlation coefficients between the independent variables and *T*_peak_ measured during the 30-min work trial can be seen in Fig. 2 (left panel). Factors included in the regression analysis to explain the variance in *T*_peak_ were A_D_:mass, log(E_2_), *T*_0_ and power output. The decision to enter A_D_:mass was made as it is a function of both individual factors and that it provided the strongest correlation to *T*_peak_, while *T*_0_ (but not *T*_base_) was entered to reduce collinearity and because it provided far stronger correlation to *T*_peak_. The resulting model can be seen in Table 4, with no evidence of serial correlation in the model (2.15), and very high tolerance values indicating acceptable collinearity and model stability. Variables that were excluded from the models were A_D_:mass (*β* = 0.08, *p* = 0.26). Overall, the model was able to account for 60% of the variance in *T*_peak_, with *T*_0_ the largest contributing variable (Fig. 2, right panel). It is noteworthy that the resulting model remained unchanged even when the omitted variables (*A*_D,_ mass and *T*_base_) were included a posteriori, supporting the decision process.

**Table 4:** Multiple regression models for explaining the core temperature response (*T*_peak_) and performance (mean power output)

	*B*	95% CI	*β*	*p*	Tolerance	$\overline{R }$^2^(%)
*T*_peak_						
Constant	4.10	− 2.49–10.68		0.22		
* T*_0_, °C	0.89	0.71–1.06	0.65	< 0.01	0.98	41.1
Power, W	0.01	0.00–0.01	0.41	< 0.01	0.99	14.9
log(E_2_)	0.12	0.04–0.19	0.20	< 0.01	0.98	3.5
Power output						
Constant	− 34.20	− 77.99–7.24		0.11		
PPO, W	0.40	0.30–0.51	0.58	< 0.01	1.00	34.7
RPE	4.60	2.43–6.77	0.31	< 0.01	1.00	9.2

*B* unstandardized regression coefficient, 95% CI confidence intervals of the slope coefficient or intercept, *β* standardized regression coefficient, $\overline{R }$^2^ adjusted partial contribution to total variance

### Power output

Correlation coefficients between the independent variables and mean power output achieved during the 30-min work trial can be seen in Fig. 4 (left panel). Factors included in the regression analysis to explain the variance in power output were *A*_D_, VO_2_max, PPO, training history, WBSR and RPE. The resulting model can be seen in Table 4, with no evidence of serial correlation in the model (1.86), and very high tolerance values indicating acceptable collinearity and model stability. Variables that were excluded from the models were *A*_D_ (*β* = − 0.03, *p* = 0.72), VO_2_max (*β* = 0.16, *p* = 0.11), training history (*β* = 0.09, *p* = 0.22), and WBSR (*β* = 0.10, *p* = 0.24). Overall, the model was able to account for 44% of the variance in power output, with peak aerobic power the largest contributing variable (Fig. 4, right panel).

## Discussion

The current study fills an important gap in the literature that describes a woman’s vulnerability to exertional heat stress in this literature. Namely, it is the first study to determine the relative contribution of independent variables (individual factors) in explaining the core temperature response to exertional heat stress in women *at very high metabolic rates*, and *when accounting for the inter- and intra- variation in ovarian hormone concentrations* (cf. Havenith et al. 1998; Notley et al. 2019). In partial support of our hypothesis, we observed that E_2_ contributes a small amount toward the core temperature response (*T*_peak_), whereby starting core temperature and power output (≈metabolic heat production) explained the greatest variance.

**Fig. 3:**
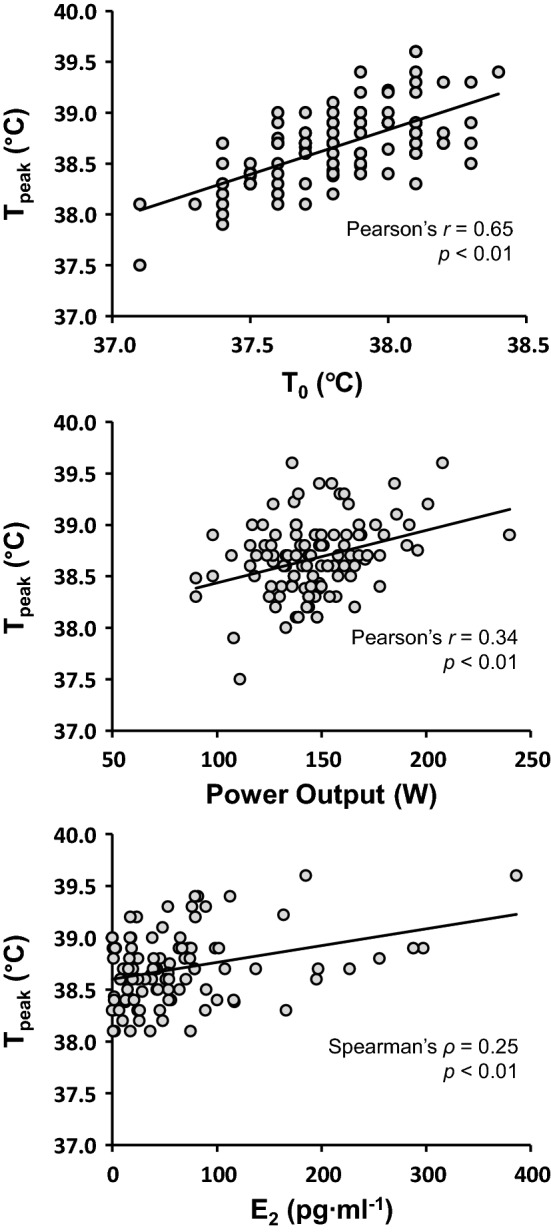
Bivariate associations between peak core temperature (*T*_peak_) during exercise and core temperature at the start of the work trial (*T*_0_; top row, *n* = 115); between *T*_peak_ and mean power output during the work trial (middle row, *n* = 115); between *T*_peak_ and E_2_ concentration measured before exercise (bottom row, *n* = 104). Values are all common individual data points, analyzed using Pearson’s correlation coefficient and Spearman’s rho, respectively

In the current study, E_2_ was positively associated with *T*_peak_, although it was only able to explain ≤ 4% of its variance (Fig. 2, Table 4). This seemingly contradicts other research (Charkoudian and Stachenfeld 2014) and is inconsistent with our previous findings. A subset of these results (Lei et al. 2019) showed that the OCP group had attenuated heat loss mechanisms (↑ forearm vascular resistance, ↓ forearm blood flow, local and whole body sweat rates) compared to their matched eumenorrheic counterparts, concurrent with lower concentrations of E_2_ (19 ± 26 vs. 78 ± 65 pg·ml^−1^; *p* < 0.01; Cohen’s *d* = 1.2), although these differences were insufficient to change *T*_core_. Furthermore, despite no change in endogenous E_2_ and P_4_, the OCP group still demonstrated a consistent and significant increase in resting and exercising *T*_core_ during their *quasi*-ML compared to EF (Lei et al. (2019). Using the current analysis (and design), it is difficult to determine whether it is the intra-participant or inter-participant E_2_ driving this relation (or both, Table 2, Fig. 3). Similarly, what modulating effect P_4_ might be contributing is unclear and is probably best explored using different methods, e.g., use of progestin-only OCP or temporary suppression of the menstrual cycle with a gonadotropin releasing hormone (ant)agonist (Charkoudian and Stachenfeld 2014). A confounding factor in this analysis may be that the group with the lowest concentrations of E_2_ was younger and had a lower training history (Table 1). Aerobic training, independent of aerobic fitness (VO_2_max), has been shown to improve *T*_core_ and heat loss responses in both men (Ravanelli et al. 2021) and women (Ichinose et al. 2009) synonymous with phenotypic heat adaptation. Clearly, further research on this topic is necessary in additional cohorts (e.g., ages and training status); nevertheless, the effect of E_2_ on *T*_peak_ was still considerably less than that of starting *T*_core_ and power output.

That *T*_0_ was able to explain ~ 40% of the *T*_core_ response should reinforce for women what is already known and practiced for men with regard to heat-specific interventions; namely, trained women should focus and prioritize interventions (e.g., aerobic training, active heat adaptation, pre-exercise cooling, fluid ingestion etc.) that effectively lower *T*_core_ before competition, attenuate the rise in *T*_core_ during or (perhaps) extend *T*_core_ at the end of exercise in order to improve work output (Alhadad et al. 2019). Moreover, power output explained ~ 15% of the *T*_core_ response, which reaffirms the contribution of metabolic heat production (Nielsen 1938; Notley et al. 2019). This highlights the role that behavioral thermoregulation (self-pacing) plays during exercise in the heat by being able to reduce metabolic heat production, thereby improving heat exchange with the environment to decrease thermoregulatory strain, something that a fixed-intensity protocol does not permit (Schlader et al. 2011a, b, c).

**Fig. 4:**
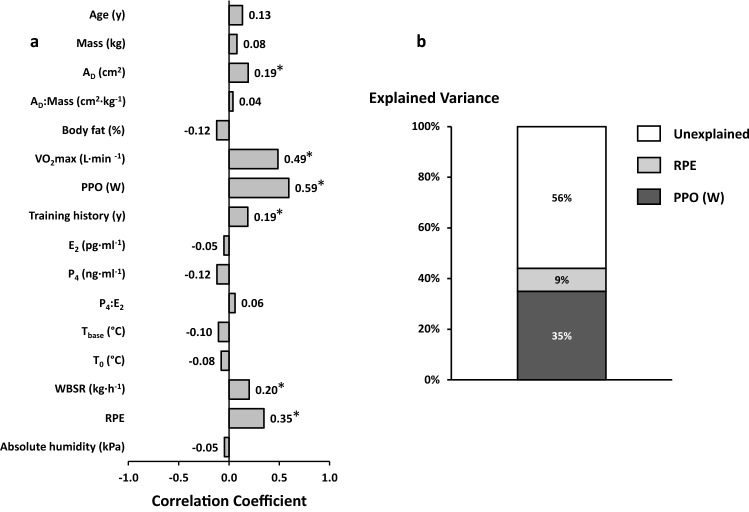
**a** Bivariate associations between independent variables and mean power output on all common data points. *p < 0.05. **b** The percentage of explained and unexplained (residual) variance ($\overline{R }$^2^) for explaining mean power output

Few studies have previously quantified contributors to aerobic performance during self-paced exercise in the heat; to the authors’ knowledge, this is the first study to do so using women. The single greatest contributor toward work output (performance) was a participant’s peak aerobic power (Fig. 4, Table 4). These results support those of James et al. (2017) who demonstrated that velocity at VO_2_max (i.e., PPO) was the strongest predictor of 5-km running performance in the heat in men. Thus, the results of the current study and James et al. (2017) concur with a recent meta-analysis (Alhadad et al. 2017) that placed aerobic training as the single greatest factor for determining endurance performance in the heat, above heat acclimation, pre-exercise cooling and fluid ingestion, something that athletes and practitioners should consider.

Notable differences between our results and those previously (Havenith et al. 1998; Notley et al. 2019) include: (i) anthropometric factors such as body mass and A_D_ (or composite, mass:*A*_D_) did not contribute toward variance explained in *T*_peak_ despite significant correlations (Fig. 2); (ii) the functional factor of VO_2_max did not contribute toward variance explained in *T*_peak_ (Fig. 2), and although it correlated with power output, it did not contribute toward variance explained (Fig. 4); (iii) the environmental factor of vapor pressure did not contribute toward variances explained (Figs. 2 and 4). As already mentioned, we believe these differences to be likely a function of the different sample training status and protocol used (intensity and self-pacing). However, it is also acknowledged that like other retrospective analyses of existing datasets (Havenith et al. 1998; Notley et al. 2019), the current analysis has certain limits. Our primary focus was whether and by how much the *T*_core_ response to exertional heat stress in women can be explained by accounting for the variation in ovarian hormone concentrations. To maximize predictive/explanatory power, we chose to include all factors into one model each for power output and *T*_peak_, i.e., by not separately grouping by vapor pressure, pre-load duration, etc. Thus, due to our partially nested design, we cannot be certain of the independent effect of these variables. Nevertheless, if we were to take by example the dependent and independent variables with greatest explanatory power (*T*_peak_, power output, *T*_0_, RPE) and compare between vapor pressures and pre-load duration, no differences are found (all *p* > 0.21). Furthermore, were the factor of vapor pressure to exert an effect, then this should be evident as a positive (*T*_peak_) or negative (power output) correlation, which is not evident in our results (Figs. 2 and 4). Moreover, it is noteworthy that the resulting models (± 1–6%) and predictors remain largely unchanged if vapor pressure and pre-load were separated.

### Considerations

The observations herein are valid only for the current sample(s), protocol(s) and condition(s), and inference of association does not imply causation. It is regrettable that measurement of autonomic thermoeffectors and thermodynamic data were not collected in ~ 40% of the sample, which may have strengthened the results. Our decision to use *T*_peak_ as our primary dependent variable was guided by the fact that (i) ethics committees and professional bodies use absolute, not relative, thresholds for *T*_core_ in their guidelines and policies; ii) not all participants reached their highest *T*_core_ at the end of exercise due to the self-paced nature of the protocol. However, a posteriori re-analysis of our data for ∆* T*_core_ did not change any of the significant independent variables. While it may be tempting to interpret the results as E_2_ having a negligible influence on *T*_core_/*T*_peak_, it is worthwhile considering that as an individual factor E_2_ did contribute a small amount toward the variance explained for *T*_peak_, whereas *A*_D_:mass did not, a variable that has previously been shown to have one of the largest effects (Havenith et al. 1998). Finally, our data should not be generalized to other OCP formulations (e.g., triphasic combination and progestin-only) or to the late-follicular/pre-ovulatory phase of a menstrual cycle.

### Perspectives and significance

Women remain underrepresented in the exercise thermoregulation literature and > 70% of studies still *do not* report ovulatory status or menstrual phase (Hutchins et al. 2021). Ovulatory status should not inhibit inclusion into this research topic (Schaumberg et al. 2017; Zheng et al. 2021b) although, importantly, the current results support calls for future measurement and consideration of ovarian hormone concentrations being standard (Elliott-Sale et al. 2021). Individualization of human thermoregulation models improves the prediction of heat strain, largely through an increase in the number of input parameters (Havenith 2001). The current results suggest an additional factor (E_2_) might be considered in future work, although data saturation has not been reached. Similarly, Flouris et al. (2018) have identified simple metrics that can successfully be used as screening criteria to prospectively identify individuals at greater risk of acute exertional heat stress. Flouris et al. (2018) argue health professionals and occupational management to (re)consider whether different criteria for women should be utilized on account of their unique body morphology/physiology, something the current results support.

## Abbreviations

A_D_: Du Bois body surface area
ANO: Anovulatory group
DRY: Warm–dry environment
E_2_: 17-β Estradiol
EF: Early follicular
HUM: Warm–humid environment
ML: Mid-luteal
OCP: Oral contraceptive pill
OVU: Ovulatory group
P_4_: Progesterone
$\overline{R }$^**2**^: Adjusted partial contribution to total variance
RPE: Rating of perceived exertion
*T*_0_: Core temperature at start of work trial
*T*_base_: Core temperature at baseline
*T*_core_: Core temperature
*T*_peak_: Peak core temperature
*T*_rec_: Rectal temperature
VO_2_max: Maximal O_2_ uptake
WBSR: Whole body sweat rate
